# Design, assembly, and validation of a nose-only inhalation exposure system for studies of aerosolized viable influenza H5N1 virus in ferrets

**DOI:** 10.1186/1743-422X-7-135

**Published:** 2010-06-23

**Authors:** Richard S Tuttle, William A Sosna, Deirdre E Daniels, Sara B Hamilton, John A Lednicky

**Affiliations:** 1 Aerosol Research and Engineering Laboratories, 13300 West 98th Street, Lenexa, Kansas, 66215, USA; 2 Energy and Life Sciences Division, Midwest Research Institute, 425 Volker Boulevard, Kansas City, Missouri 64110, USA

## Abstract

**Background:**

The routes by which humans acquire influenza H5N1 infections have not been fully elucidated. Based on the known biology of influenza viruses, four modes of transmission are most likely in humans: aerosol transmission, ingestion of undercooked contaminated infected poultry, transmission by large droplets and self-inoculation of the nasal mucosa by contaminated hands. In preparation of a study to resolve whether H5N1 viruses are transmissible by aerosol in an animal model that is a surrogate for humans, an inhalation exposure system for studies of aerosolized H5N1 viruses in ferrets was designed, assembled, and validated. Particular attention was paid towards system safety, efficacy of dissemination, the viability of aerosolized virus, and sampling methodology.

**Results:**

An aerosol generation and delivery system, referred to as a Nose-Only Bioaerosol Exposure System (NBIES), was assembled and function tested. The NBIES passed all safety tests, met expected engineering parameters, required relatively small quantities of material to obtain the desired aerosol concentrations of influenza virus, and delivered doses with high-efficacy. Ferrets withstood a mock exposure trial without signs of stress.

**Conclusions:**

The NBIES delivers doses of aerosolized influenza viruses with high efficacy, and uses less starting material than other similar designs. Influenza H5N1 and H3N2 viruses remain stable under the conditions used for aerosol generation and sample collection. The NBIES is qualified for studies of aerosolized H5N1 virus.

## Background

Human infections caused by highly pathogenic avian influenza H5N1 viruses (H5N1) that arose from 2003-onwards have been rare (495 cases confirmed through April 21, 2010) but have a fatality rate of about 59% [1]. There is limited knowledge about the potential routes and determinants required for H5N1 transmission to and between humans. Human-to-human transmissions have rarely been reported, and have been limited, inefficient and un-sustained. In ferret transmission models, H5N1 are inconsistent in transmission by direct or indirect contact exposure, but direct intranasal exposure causes morbidity and sometimes, mortality (2, 3, and J. Lednicky, unpublished). In contrast, the 1918 pandemic influenza virus was easily transmissible human-to-human, and caused the deaths of between 20 - 40 million people worldwide for a lethality rate of 2.5%. Whereas the differences in transmissibility and lethality between the two viruses are not fully understood, performing well-controlled inhalation exposure studies of aerosolized viable H5N1 in appropriate animal models may improve our understanding of factors responsible for -the acquisition of H5N1 infections by humans and the virulence/lethality relative to route of transmission.

Four modes are most likely for the transmission of influenza viruses: aerosol transmission, ingestion of undercooked contaminated infected poultry, transmission by large droplets, and self-inoculation of the nasal mucosa by contaminated hands. Various publications state that large-droplet transmission is the predominant mode by which infection by seasonal influenza A viruses is acquired by humans [4-7], while others refer to aerosols as an important mode of transmission for influenza [8-12]. Transmission may also occur through direct contact with secretions or fomites with oral, conjunctival and nasal mucus membranes because the virus can remain infectious on nonporous dry surfaces for ≤48 hours [13]. To date, transmission of H5N1 to humans has occurred primarily through close contact with infected birds or, in a single case, consumption of raw infected duck blood [14]. There is some evidence for limited human to human transmission of H5N1 [14-18]. Transmission of influenza viruses by large droplets without accompanying aerosols has been simulated by intranasal droplet infection [19] and it is assumed that H5N1 infections may be acquired through droplet transmission routes, since intranasal inoculation of ferrets with H5N1 strains (used as a model for droplet infection) can result in clinical signs of severe influenza (3, 20, 21, 22, 23 and J. A.Lednicky, unpublished). A basic understanding of how H5N1 is transmitted to humans, and person-to-person, is valuable from a public-health perspective, not only for establishing measures to protect community health, but also for the management of hospitalized patients.

Until the time of this work, it was not clear whether humans could be infected through inhalation of aerosolized contemporary H5N1 particles. Based on the natural biology of influenza viruses, we hypothesized that clinically apparent infections could arise from inhalation of aerosolized H5N1 viruses, and planned to test our hypothesis using inhalation exposure studies of aerosolized H5N1 in a ferret model. Here, aerosols are defined as suspensions of small solid or liquid particles (in air) that remain airborne for prolonged periods of times due to their low settling velocity [6,24]. The settling velocity in still air can be calculated using Stokes' law [25], and the smaller the particle, the longer the settling time.

There are two important considerations in studies of bioaerosols generated by human subjects [12]. First, it is important to distinguish between the initial diameter of particles and the diameter after evaporation of water in ambient air [the resulting desiccated particles are termed 'droplet nuclei'; for particles with an initial diameter <20 μm the evaporation occurs in <1 s [26,27] and the diameter shrinks to a little less than half the initial diameter [26]]. The second important consideration is penetration of the respiratory tract by aerosolized particles. Particles 5 μm or less in aerodynamic diameter have a significant penetration into the human respiratory tract all the way to the alveolar region (30% penetration for 5 μm particles); particles ≥ 6 μm are increasingly trapped in the upper respiratory tract [24,25,28]. Evaluation of particle sizes are especially important for aerosol studies with influenza virus: whereas human influenza viruses specifically recognize α2,6-linked sialic acid (SA) receptors, which are dominant on epithelial cells in the upper respiratory tract [29], contemporary H5N1 affecting humans specifically recognize α2,3-linked SA receptors, which are located in the lower respiratory tract [29,30]. These receptors are not easily reached by the large droplets (diameter of >10 μm) typically produced by coughing or sneezing [4]. Noteworthy, penetration is not the same thing as deposition. Due to a myriad of variables, only a fraction of the penetrating particles may be deposited, the remainder exhaled back [24,31].

Because some of the circulating H5N1 have demonstrated uncharacteristic affinity for α2,6-linked SA receptors and are therefore potentially dangerous to humans [32,33], it is important to evaluate their airborne transmissibility in a suitable animal model. Domesticed ferrets (*Mustela putorius furo *) have respiratory tracts that share many anatomic features with those of humans, and have metabolic and physiologic similarities with humans. They are an appropriate animal model [34] for study of the pathogenicity [20,35] and transmissibility [36,37] of influenza viruses. On the basis of H5N1 virus cell tropism in their lower respiratory tract, ferrets have also been used as a small animal model of human H5N1 pneumonia [30].

In preparation for studies of the transmissibility of aerosolized H5N1 particles, we developed a nose-only inhalation exposure system (NBIES) that can be used to conduct studies with H5N1 and other influenza viruses in ferrets (or other small animals). The NBIES configuration described here produces aerosolized particles with diameters appropriate for deep lung penetration. The assembly and validation of the system are described in this manuscript.

## Results

### 1. NBIES overview

**Figure 1:**
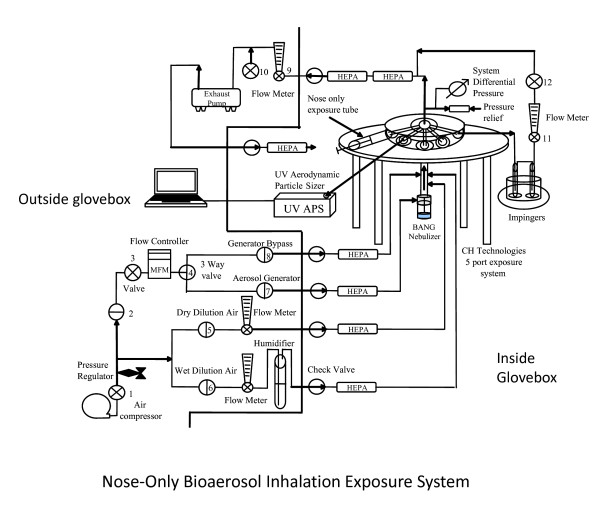
**Schematic representation of the NBIES**. Components outside (left) and inside (right) the glovebox are demarcated.

The NBIES is comprised of aerosol generation equipment, animal holders, samplers, a class III glovebox, and the pumps, flow meters, and other equipment required for the control, balancing, and measurement of airflows and aerosols. It is housed within an ABSL3-enhanced laboratory. A schematic diagram of the NBIES is shown in Figure 1. A nose-only system design was chosen over whole-body and other exposure system designs for the NBIES for the following reasons: (1) It minimizes the establishment of infection by non-inhalation routes. The probability of acquiring infection through non-inhalation routes, such as ingestion of test agent deposited in fur during whole-body exposure studies, is significantly higher using other exposure systems, (2) It reduces the requirements for post-exposure decontamination of animals. Otherwise, it is common practice to decontaminate an animal with a bleach (or similar) solution during inhalation exposure studies using other types of dissemination devices, (3) It reduces potential contamination of animal housing areas and animal care personnel. (4) It permits testing with high concentrations of aerosolized agent while minimizing quantities of starting material. This is a useful feature when working with select agents in the USA, since their production and destruction are performed following a cradle to grave documentation process, and due to restrictions on the amounts of agent that can be produced. To eliminate potential release of aerosolized agent into the test facility, the exposure system is operated under negative pressure relative to that of the glove box, which is operated at negative pressure relative to the laboratory. System components located outside of the class III glovebox are routed through a bulkhead panel using a series of one-way (check) valves and HEPA filters attached in series.

### 2. Operational description

The NBIES functions in the following manner: high-pressure air generated by an air compressor provides both supply air for the generation of aerosols -and "dilution" air used to create the desired aerosol concentration and flow. Air supplied to the system is controlled at precise flow rates and pressures with electronic and manual valves and is metered using mass flow meters and controllers. As air is supplied to the aerosol generator, a high velocity air stream creates a venturi effect that siphons liquid through a tube from the nebulizer reservoir that contains the virus (or other agent) suspension. As the liquid exits the tube at the top of the nebulizer, the air stream interacts with the liquid and shears it creating a gas-phase micron-sized aerosol stream. The size of the resulting aerosol particles is a function of the air velocity regulated by the air supply pressure and flow rate, and the volumetric use rate of the nebulizer. From the nebulizer, a minimally polydispersed aerosol stream travels into an exposure system delivery tube, where additional filtered dilution air is introduced upstream of the exposure system to supply additional air for animal respiration, sampling systems, humidity conditioning and control of the aerosol challenge concentration. Ferrets are housed in restraint tubes attached to the delivery ports of exposure system and are positioned such that their muzzles are in close proximity to the ports from which the aerosol stream is delivered for inhalation. The system is operated in a dynamic (not static) mode; the aerosol stream passes through the inhalation ports and exhausted through an outer plenum of the exposure system. The exhaust system consists of a valved exhaust pump equipped with HEPA filters. The exhaust flow rate is regulated to maintain a continuous negative pressure within the exposure system relative to the class III glovebox and is monitored using a Magnehelic differential pressure gauge. The system is operated at air supply flow rates sufficient to provide a continuous regeneration of fresh aerosol- stream to the animals, reducing potential aerosol-stream and carbon dioxide re-breathing concerns. Two of the exposure ports are utilized during exposure challenges for real-time measurement of aerosol particle size, and collection (sampling) of aerosolized particles for viability counts. The size distribution of aerosolized particles is measured using an Aerodynamic Particle Sizer^® ^Spectrometer (APS), and viable agent exposure challenge and dose concentrations are determined from the quantity of viable test agent that is collected within impingers.

In the challenge-material preparation process, influenza virus is mixed with a non-toxic delivery vehicle (sterile PBS solution with 0.5% purified BSA fraction V) to help maintain viability of the virus and act as a vehicle to generate the aerosol stream. The saline solution is well characterized and its acute inhalation toxicity known (it does not cause an acute inflammatory response when inhaled by ferrets in the quantities used in this work). An antifoam agent is added to the starting material and collector fluids in the impingers to reduce bubbling.

### 3. Validation of system engineering and function

**Figure 2:**
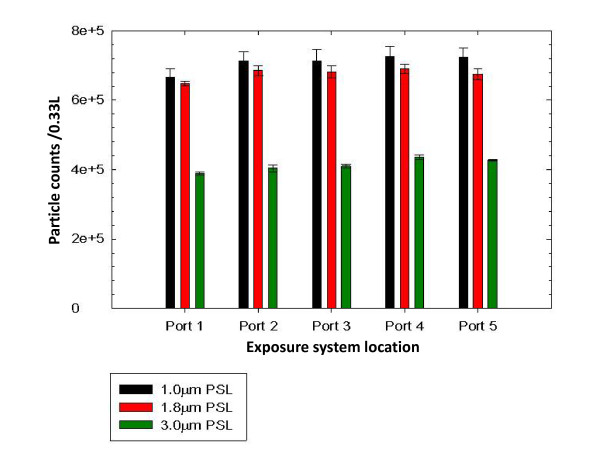
**Aerosol exposure port to port homogeneity plot**. Total particle counts for three different-sized PSL microspheres in an aerosol volume of 0.33 L (with a read time of 20 sec) are shown. The results shown are for three separate determinations.

Initial operating parameters of the exposure system were based on ferret respiratory requirements, and sampling system, particle size monitoring, and generation-system flow-rate requirements, A commercial non-ionic detergent solution (Snoop Leak Detector, Swagelok Co., Solon, OH) was used to detect leaks in the air-handling and system components after assembly of the NBIES. Leaks detected (if any) were sealed and the system repeatedly retested to verify remediation. To improve safety and extend durability, plastic tubing and valves were replaced with stainless steel equivalents where possible. During the pre-study development phase, evaluation of the NBIES included measurements of exposure port-to-port aerosol concentration homogeneity, concentration increase over time, aerosol concentration stability over the exposure duration, and decline over time, which were evaluated using calibrated National Institutes of Standards and Technology (NIST) - traceable polystyrene latex (PSL) microspheres of three different sizes. For these assays, measurements were taken using the APS. Figure 2 (relevant data points in Table 1) shows a plot representing the aerosol exposure port-to-port homogeneity comparing the aerosol concentration uniformity of different-sized PSL microspheres delivered to each exposure port (results of three separate trials). These experiments to assess aerosol homogeneity did not require uniform starting concentrations of the three different-sized PSL suspensions; thus the total counts (aerosol concentration levels) for the different-sized PSL suspensions vary in Figure 2 but are relatively uniform within a microsphere size group. The slight variability (lower counts) for port 1 (Figure 1 and Table 1) are due to the time lag when moving the APS probe to port 1 in the glovebox, and are not due to system design failure or problems.

**Table 1:** Five minute aerosol characterization results for A/Wisconsin/67/2005 (H3N2).*

**Virus Conc. (TCID**_**50**_**/ml) in BANG reservoir**	**Virus quantity in impinger A (TCID**_**50 **_**units)**	**Virus quantity in impinger B (TCID**_**50 **_**units)**	**Theoretical 100% recovery (TCID**_**50 **_**units)**	**Approximate collection efficiency impinger A**
4.6 × 10^6^	83 750	BLD**	92 000	91.0%
4.6 × 10^6^	82 860	BLD	92 000	90.1%
4.6 × 10^6^	89 990	BLD	92 000	97.8%
4.6 × 10^5^	8 732	BLD	9 200	94.9%
4.6 × 10^5^	8 197	BLD	9 200	89.1%
4.6 × 10^5^	8 464	BLD	9 200	92.0%

*Running conditions: nebulizer use rate, 0.1 ml/min; generation time, 5 min; system total flow rate, 5 L/min; impinger sample rate, 1 L/min; impinger sample time, 5 min.

**BLD, below limits of detection.

**Figure 3:**
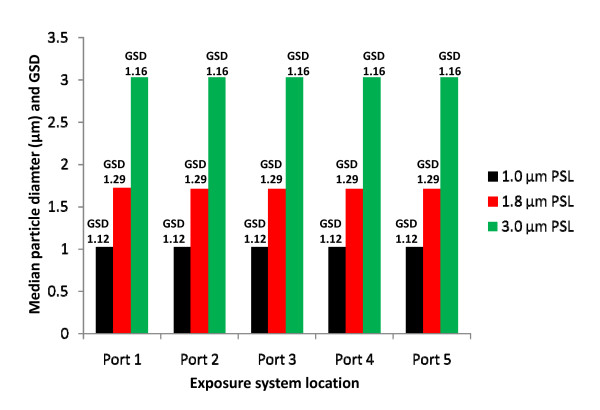
**Microsphere size distribution homogeneity characterization plot**. The median particle diameters and GSD are shown for three different-sized PSL microspheres.

Figure 3 depicts the results of size distribution homogeneity characterization tests; measurements of both number median diameter and geometric standard deviation (GSD) are shown. The GSD is a measure of dispersion for a log-normal distribution and is analogous to the standard deviation for a normal distribution. It is the ratio of the 84.13 percentile to the 50 percentile, and aerosols with GSD >1.2 are considered to be polydispersed (i.e., the particles vary significantly in size). As shown in Figure 3, the 1.8 μm particles, with a GSD of 1.29, are nominally polydispersed; the 1.0 and 3.0 μ microspheres are nearly monodispersed (nearly unimodal). With the GSD in the range of 1.2, and the median values indicating a size distribution that centers on the PSL manufacturer's specifications, this characterization data is acceptable since it suggests the system is functioning as desired. The data shows the port to port size distribution and GSD's of select aerosol particle sizes generated from suspension do not differ from port to port on the aerosol system. The results indicate that aerosols generated from the nebulizer (at the flow rates used for the NBIES for exposures) deliver a dry and uniform particle size to each of the NBIES' five ports. Therefore, inhalation (and presumably lung deposition) of the generated aerosolized agent should be similar for animals at any of the five locations.

**Figure 4:**
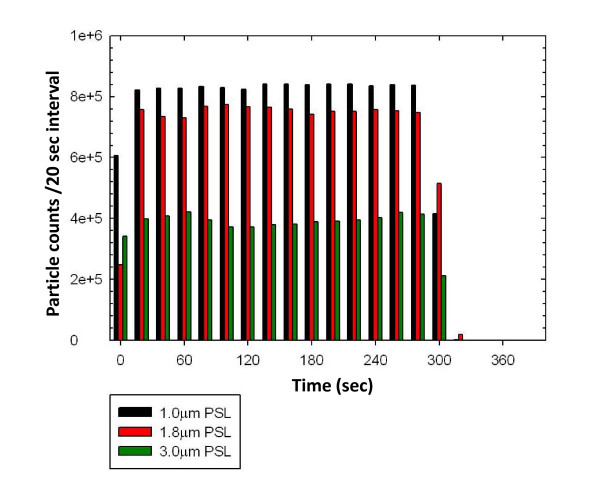
**Port to port concentration vs time profile**. Counts were taken from a sampled volume of 0.33 liters of aerosol stream at 20 sec intervals.

Figure 4 depicts aerosol concentration trends for 5 min characterization tests of the NBIES with three sizes of uniform polystyrene microspheres; rapid ramp-up, stability of aerosol concentration during dissemination, and rapid decline at termination are evident. Evaluating these properties of the aerosol system is important; deviations from homogeneity affect accuracy during the delivery of exposure doses, and the performance of reproducible exposure trials.

### 4. Evaluation of aerosol vehicle

Based on cumulative experience (J. Lednicky, unpublished), an aerosol vehicle comprised of (PBS + 0.5% BSA + antifoam agent media) was prepared and tested. Influenza virus is suspended in the aerosol vehicle (which is designed to maintain the virus' viability for several hours at room temperature), and the suspension added to the nebulizer reservoir prior to creation of the aerosol stream that disperses and delivers the virus. Virus viability was shown stable at room temperature over a period of three hours in the aerosol vehicle (data not shown).

Thereafter, the aerosol size distribution of the aerosol vehicle was characterized. Tests indicated a particle size distribution including a range needed for deep lung penetration (data not shown). The PBS + 0.5% BSA + antifoam agent media was also utilized as the aerosol-stream collection media for impinger samplers.

### 5. System integrity and safety test using live agent

A safety test was performed as part of the system validation process with live-agent dissemination of *Influenza virus *A/Wisconsin/67/2005 (H3N2) [Wis/05]. No releases were detected by environmental sampling for live agent (data not shown).

### 7. Particle size analyses of aerosolized Wis/05

**Figure 5:**
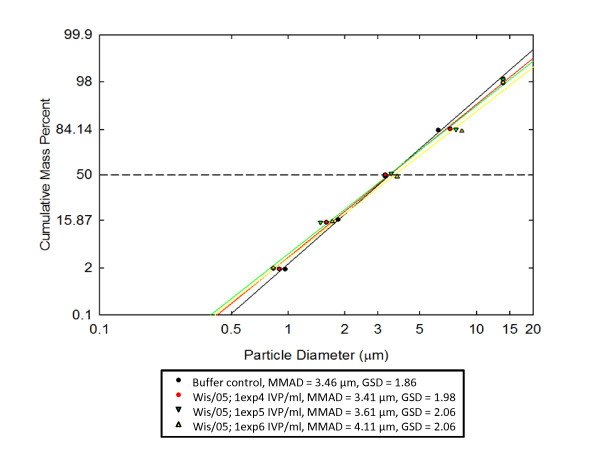
**Aerosol size log-probability plot for Wis/05**. The MMAD and GSD are indicated at three different concentrations of virus and for the control solution.

Wis/05 was aerosolized starting with three different concentrations of virus, and the sizes of the particles generated were analyzed. The results are depicted in an aerosol particle-size log-probability plot in Figure 5. The mean mass aerodynamic diameter (MMAD), which is the diameter that divides the frequency of particles in half, ranges from 3.41 to 4.11 μ, indicating a significant proportion of particles of the size needed for deep lung penetration (≤5 μ) were present.

### 8. Evaluation of impinger performance

Precision in calculations of aerosol concentrations and estimates of the number of viruses inhaled per experiment depend largely on the collection efficiency/efficacy of the sampler(s). Therefore, the sampling system must first be characterized to establish operational parameters determined to obtain a presented dose (*D *) (defined as the inhaled dose estimated from the multiplication of the aerosol concentration and the total volume of air breathed in by an animal). *D *is estimated from an animal's respiratory rate and the duration of its exposure to aerosolized agent. Systems similar to the NBIES are often designed with a single impinger and are operated with the assumption that > 90% of the aerosolized microorganisms are entrained (trapped) during sampling of the aerosol flow through the sampler, and that collection is representative of the viable inhaled dose and retention in the animal model. If the efficiency of the collector is not known, a significant undercount of the aerosol concentration can result, causing both an underestimate of the inhaled dose and an overestimate of virulence (since the number of organisms to cause an infection is undercounted). Moreover, the collection fluid in the impinger must maintain the aerosolized agent in a viable (infectious) manner and quantification should be for viable agent. Otherwise, quantification of aerosolized agent based solely on biochemical or immunological assays (such as PCR or ELISA) may complicate and confound understanding by measuring both live and inactivated agents. Although the effect of the collection process on the viability of the entrained agent in the impingers can only be inferred, collection efficency of our sampling system using fundamental mathematics could be derived. The NBIES was designed with a dual impinger arrangement based on previous experience (Richard Tuttle, unpublished). The impingers selected were low collection flow, low velocity collectors that apply minimal collection forces (e.g. impaction and turbulence) on the aerosolized agent during the collection process. Thus, they reduce the impact of compromising the viability of the collected organism in relation to other collectors that are typically used in bioaerosol studies. By using a dual impinger collection system with primary and secondary impingers sampling in-parallel and in-series, it was discovered that aerosolized *Influenza virus *A/Vietnam/1203/2004 (H5N1) [VN/04] was not collected with high efficiency with one impinger alone under the conditions used, whereas Wis/05 was largely entrained by one impinger (results for 5 min test, Table 1). In contrast, VN/04 was not entrained effectively by the primary impinger alone (examples shown in Tables 2 and 3). A possible explanation is that primary isolates of influenza viruses are often filamentous, whereas laboratory strains appear more spherical to ovoid, and thus their physical characteristics are somewhat different. The H5N1 strain used in this work is filamentous. Once a virus is aerosolized and "dry", it may become hydrophobic and hard to rehydrate, decreasing collection in impinger media, and it may be harder to rehydrate a long filamentous influenza virus particle. In support of this notion, there was no detectable virus in impinger B when impinger A was spiked with VN/04 and the NBIES operated for 5 or 10 min (data not shown).

**Table 2:** Five minute aerosol characterization results for A/Vietnam/1203/2004 (H5N1).*

**Virus Conc. (TCID**_**50**_**/ml) in BANG reservoir**	**Virus quantity in impinger A (TCID**_**50 **_**units)**	**Virus quantity in impinger B (TCID**_**50 **_**units)**	**Theoretical 100% recovery (TCID**_**50 **_**units)**	Approximate collection efficiency impingers A + B
4 600 000	10 450	9 975	92 000	22.2%

*Running conditions: nebulizer use rate, 0.1 ml/min; generation time, 5 min; system total flow rate, 5 L/min; impinger sample rate, 1 L/min; impinger sample time, 5 min.

**Table 3:** Ten minute aerosol characterization results for A/Vietnam/1203/2004 (H5N1) with three impingers.*

**Virus Conc. (TCID**_**50**_**/ml) in BANG reservoir**	**Virus quantity in impinger A (TCID**_**50 **_**units)**	**Virus quantity in impinger B (TCID**_**50 **_**units)**	**Virus quantity in impinger C (TCID**_**50 **_**units)**	**Theoretical 100% recovery (TCID**_**50 **_**units)**	Approximate collection efficiency impingers A+B+C
4 600 000	19 665	19 665	10 165	184 000	26.9%

*Running conditions: nebulizer use rate, 0.1 ml/min; generation time, 10 min; system total flow rate, 5 L/min; impinger sample rate, 1 L/min; impinger sample time, 10 min.

### 9. Sham run with ferrets

Ferrets were tested using sham 10-min runs. No problems were encountered; there was no evidence of heat stress, or indications of improper airflow control (such as cyanosis resulting from CO_2 _build-up). No reddening of the skin (another indicator of stress) was detected. Ferrets did not seem agitated upon release from the exposure system and rapidly resumed normal activity.

## Conclusions

The data trends shown in Figures 2 to 4 depict desirable results for an aerosol system that delivers reproducible and accurate aerosol challenges with precision and accuracy. The data shows uniform delivery of aerosol concentration at each exposure-location, rapid increase to stable-state concentration, stable aerosol delivery over time, and a rapid decrease or purge of the aerosol from the system after the exposure challenge is terminated.

Doses of influenza viruses were delivered at efficiencies ranging from 9 - 98% (sample data shown in Tables 1, 2, and 3). Due to a paucity of data, direct comparisons of the delivery of influenza viruses with other systems are difficult. However, similar systems typically deliver various types of doses with lower efficiencies, such as ~0.05% for a one-jet Collison nebulizer at an air pressure of 5 psig [38]. Thus, the NBIES is highly effective. Experiments with VN/04 clearly indicate that an undercount of the aerosolized virus can occur if only one impinger is used. Two or more impingers should be used for accuracy/precision. Otherwise, incorrect estimates of delivered dose and analyses of system efficiency occur.

The particle size measurements showed consistent aerosolized particle delivery (for all four dose groups) that centered on a size range that should be respired and deposited in the lower respiratory tract of humans. There was little difference in particle size-distribution and median diameter of the buffer control alone in comparison to aerosolized virus. This suggests the possibility that an influenza virion can be encapsulated within the salt-BSA complex without greatly affecting the overall dimension of the aerosolized particle. However, there is no formal proof that the particles detected by the APS indeed contained virus (the virus may have aerosolized as free virus particles with size below the detection limit of the instrument).

The NBIES exposure port flow velocity (0.234 m/s) is relatively low (~0.52 m/hr or ~0.84 km/hr); therefore undue stress caused by airstream velocity in the region of the animal's muzzle is not an issue. The volume of air space in front of the animals nose (approx. 12.9 ml) is small and changes frequently relatively to the volume of air delivered/min for each port (*Q *_*port *_); thus, re-breathing of exhaled air and old (residual) aerosolized viral particles should not occur. The system flow rate *Q *_*sys *_of 5 L/min surpasses the calculated *V *_*m *_for 5 animals by a factor of about 2.9× with a high estimate of 0.345 L/min for *V *_*m *_, and a factor of 5× with a value of 0.2 L/min. The same values apply to air changes; at 0.345 L/min, the number of air changes required is 0.345 L/min × 5/0.101 L = ~17.1, since there are 49.4 changes/min, ~2.9 air changes occur, showing that more than adequate airflow is supplied to the system for respiration and replenishment of fresh aerosol in the respiration zone of the animal. Adequate air flow is important for performing accurate inhaled dose calculations as well as for the reduction of stress due to the inhalation of increased CO_2 _levels that occurs when air is re-breathed.

Finally, for comparison, it is also important to evaluate the effects of inhaling large particle (~10 - 20 μm) aerosols (of influenza viruses). The NBIES is inappropriate for that application; a different aerosol generator, such as a spinning-top monodisperse aerosol generator in conjunction with an appropriate delivery system, are needed for such a study.

## Methods

### 1. Class III glovebox

A class III IsoGARD^® ^Glovebox (The Baker Company, Sanford, ME) was used to house the NBIES. The class III glovebox has a High Efficiency Particulate Air (HEPA)-filtered primary chamber and a HEPA filtered pass-thru chamber.

### 2. NBIES components

Most major components of the NBIES were purchased from CH Technologies, USA, Westwood, NJ,, including the aerosol generator and delivery system, exposure systems, and ferret restraint tubes with push rods, The aerosol delivery system includes a breathing air quality Jun-Air compressor (model OF302-25BD2) for system air supply positioned outside the glovebox, and a BioAerosol Nebulizing Generator (BANG), (BGI Inc.)., Waltham, MA). The BANG is a low flow, low dead space nebulizer that is operated in the range of 1 to 4 liters per minute. It was selected over other bio-aerosol generators as an appropriate device for the aerosolization of influenza virus. Considerations for selecting the BANG included: minimal potential damage to agent, reduced clumping of virus, uniformity of droplet size distribution, and efficiency (lower use rate and volume of virus suspension than that required by similar bio-aerosol generators). The aerosol exposure system is a five-port nose-only design manufactured out of polysulfone for chemical resistance with clear plexiglass nose only ferret restraint holders. A model 3314 APS (TSI Inc. St. Paul, MN) was used with the NBIES. The APS is used to measure the aerosol size distribution, and is capable of measuring aerosols in the range of 0.3 to 20 μm. The APS is operated with Aerosol Instrument Manager software, release version 8.0.0.0 (TSI, Inc.) run with a Dell Latitude D600 computer. The exposure, generation, sampling, and particle size analysis components of the system are located inside of the class 3 cabinet.

### 3. Regulation of exposure system negative pressure

During exposure challenges, the exposure system is regulated at a negative pressure of approximately 0.05 to 0.1 inch of water, which is monitored using a magnehelic differential pressure gauge (Dwyer Instruments, Inc), with temperature in the range of 20 - 25°C and relative humidity regulated in the range of 25 to 35 percent.

### 4. Temparature and humidity measurements

The temperature and humidity within the glovebox are monitored using a model 11-661-19 digital temperature and humidity monitor (Thermo Fisher Scientific, Waltham, MA).

### 5. Monitoring and control of system flow rate

Mass flow meters (0 - 4 L/min from Dwyer Instruments, Inc., Ivyland, PA) and a mass flow controller (Alicat Scientific, Tucson AZ) are used for system flow rate monitoring and control.

### 6. Sampling system

The sampling system consists of two model 7531 midget impingers (Ace Glass Incorporated, Vineland, NJ) connected in series, with sample flow rates controlled using a valve and monitored using a 0 - 5 L/min mass flow meter. The sampler vacuum was created using a model 400-1901 Air Cadet Pump (Barnant Company, Thermo Fisher Scientific).

### 7. Exhaust system

Airflow is drawn into the exhaust system by a 1/5-hp vacuum pump (Gast Manufacturing, Benton Harbor, MI) and exhausted through two HEPA (High Efficiency Particulate Air) capsule filters (Pall Gelman, East Hills NY) connected in series.

### 8. Impinger tests

Various tests were performed (details to be presented elsewhere). Tests included entrainment testing of the of aerosolized virus in collection media as well as spike tests to determine whether virus captured in the primary impinger (A) might be re-aerosolized and captured in the secondary impinger (B).

### 9. Establishment of operational parameters unique to the NBIES

For this study, conditions were established that resulted in >90% collection of live agent in the primary impinger with the H3N2 virus. To evaluate the viable aerosol delivery efficiency and define operation parameters of the aerosol exposure system, calculations based on (theoretical) 100% efficacy of aerosol dissemination were derived using the following steps:

(1) Assuming 100% efficiency, the quantity of aerosolized virus particles (VP) for a given *C *_*s *_is calculated as:

(2) The conc. of virus in impinger A for a given *C *_*s *_is calculated.

(3) The conc. of virus in impinger B is calculated for the same *C *_*s *_in step 2

(4) The volume sampled by both impingers (*V *_*i *_) for *t *_*exp *_is calculated (for this work, 1 L/min × 10 min = 10 L)

(5) Even dissemination by the system is assumed (based on system tests) and the apparent concentration of virus (*C *_*app *_*) *in the aerosol stream is calculated as:

(6) The volume disseminated by the system (*V *_*s *_) is calculated as = System flow rate × *t *_*exp *_

(7) At 100% efficiency, the concentration of VP in the aerosol stream (*C *_*aero *_) is: VP/*V *_*s *_

(8) The true efficiency (expressed as %) of the system is: *C *_*app *_/*C *_*aero *_× 100

(9) *D *= *C *_*app *_× *V *_*m *_× *t *_*exp *_

### 10. Operational parameters

The NBIES was operated using the following parameters:

■ Exposure time (amount of time an animal is exposed to aerosolized influenza virus) = t_exp_ = 10 min

■ System head pressure (pressure supplied by Jun air compressor) = 30 psi = 206842.8 Nm^-2^

■ Total system flow rate (aerosol flow rate) over 5 ports = Qsys = 5 L/min

■ Flow rate per system exposure port = Qport = 5 L/min/5 ports = 1 L/min

■ Nebulizer pressure = P_BANG_ = 26 psi = 179263.76 Nm^-2^ (the pressure is lower than the system head pressure due to slight pressure loses that normally occur in air-handling systems)

■ Aerosol generation rate ("use rate") (volume of liquid generated by the nebulizer/time) = 0.1 mL/min

■ Relative humidity: 25 to 35%

■ Temperature: 20 - 25 C

■ Negative pressure system: 0.05 to 0.1 inches water (~0.002 to 0.004 psi, or ~12.45 to 24.9 Nm^-2^).

■ Viable sample collection (10 ml collection fluid/impinger)

■ Impinger sample flow rate = *Q *_*imp *_= 1 L/min

■ APS sampling rate *Q *_*APS *_= 1 L/min

■ Times were measured with electronic stopwatches.

### 11. Assessments of exposure system port to port aerosol concentration homogeneity

Exposure port to port concentration homogeneity (Figure 2) was evaluated in trials with NIST-traceable PSL microspheres. PSL beads with diameters of 1.09, and 1.83 μm (Polysciences, Inc., Warrington, PA), and 3.0 μm (Duke Scientific Corporation, Palo Alto, CA) were suspended in tissue-culture grade deionized H_2_O, and separately aerosolized using the BANG nebulizer. The total number of aerosolized particles detected every 20 sec by the APS was used to define aerosol concentration.

### 12. Aerosol particle size homogeneity

Aerosol particle size and concentration (Figure 3) were analyzed as for item 11 (above) using the APS.

### 13. Starting material and impinger fluid

Virus was diluted to the appropriate concentration in the aerosol vehicle (PBS + 0.5% BSA fraction V), and molecular-grade antifoam agent B (Sigma-Aldrich, Inc., St. Louis, MO) added at 0.25% (v/v). After mixing, 4 ml of the virus + antifoam material was placed in the nebulizer reservoir. Similarly, 10 ml of PBS + 0.5% BSA fraction V but with 0.5.% (v/v) antifoam agent B was placed into each impinger for aerosol collection.

### 14. Viruses

Influenza A virus VN/04 was obtained from the United States Department of Agriculture Southeast Poultry Research Laboratory (USDA SEPRL, Athens, GA). Permits necessary for the importation and work with H5N1 viruses were acquired in accordance with federal, state, and local laws. Influenza A virus Wis/05 was obtained from the Centers of Disease Control and Prevention (CDC). The identity of the viruses was established using viral genomic sequencing.

### 15. Bio-containment facilities

*In-vitro *and *in vivo *experiments with H5N1 viruses were conducted in USDA-approved BSL3 and animal biological safety level 3-enhanced (ABSL3 -enhanced) containment facilities, respectively, and required use of personal protective equipment and occupational health monitoring program.

### 16. *In-vitro *cell growth and manipulations

Pilot studies indicated that the infectivity of the viruses of this work was higher in an MRI validated *Mustela vison *(mink) lung (Mv1 Lu) cell line than in a Madin Darby canine kidney (MDCK) cell line that is used more commonly for influenza virus work (data to be presented elsewhere). The Mv1 Lu cells were propagated in Eagle's Minimal Essential Medium (EMEM) supplemented with L-Alanyl-L-Glutamine (GlutaMAX™, Invitrogen Corp., Carlsbad, CA), penicillin, streptomycin, neomycin (Invitrogen Corp.), bicarbonate, sodium pyruvate, and gamma-irradiated fetal bovine serum (HyClone, Pittsburgh, PA). The viruses were titered in Mv1 Lu cells in serum-free EMEM supplemented with bicarbonate, pyruvate, antibiotics, and L-1-tosylamido-2-phenylethyl chloromethyl ketone (TPCK)-treated mycoplasma- and extraneous virus-free trypsin (Worthington Biochemical Company, Lakewood, NJ) in 5% CO_2 _at 37°C (VN/04) or 35°C (Wis/05). The TPCK-trypsin used for this work had higher specific activity than TPCK-trypsin acquired elsewhere (data not shown), and was used at a final concentration of 0.1 μg/ml. The 50% tissue culture infectious dose (TCID_50_) were calculated by the Reed-Muench method [39].

### 17. Propagation of VN/04 in embryonating chicken eggs

VN/04 was propagated in the allantoic cavity of 10 day-old SPF *Chicken anemia virus *(CAV)-free embryonating chicken eggs (Charles River Laboratories, Wilmington, MA) [40-42].

### 18. Sham inhalation exposure of ferrets

Studies were performed using descented, spayed 3-month-old female ferrets (0.5 - 0.9 kg) (Triple F Farms, Sayre, PA). Room conditions for all work included 12 hr. light cycles, and an average relative humidity at 30% within a room temperature range between 64° and 84°F (17.8° to 28.9°C). The animals were fed pelleted ferret food (Triple F Farms) and watered *ad libitum*, and housed and maintained under applicable laws and guidelines with appropriate approvals from the Midwest Research Institute Animal Care and Use Committee. Conscious ferrets were used for the inhalation studies. Prior to performing the study, the ferrets we acclimated to the exposure tubes over a two day period. During the study, all work was performed expeditiously to minimize stress, and animals were moved in and out of the ferret restraint tubes relatively quickly. Just prior to exposure, the animals were loaded into the exposure restraint tubes and quickly transported to the class III glovebox housing the inhalation exposure system. The tubes were affixed onto designated inhalation ports on the NBIES, the aerosol challenge generated, and the animals exposed according to experimental design.

### 19. Calculations and definitions for aerosol transmission studies with ferrets

By convention used in aerobiology, where *R *refers to respiration rate, C refers to the concentration of aerosolized agent, *f(t) *= % of agent deposited in the lungs, and *t *_*exp *_*= *exposure duration time. When the following assumptions are made: a constant minute volume (V_m_) for *R(t)*, a constant live-agent aerosol concentration (integrated air sample determined concentration for *C(t)*, 100% deposition for *f(t)*, and *t(exp) *is fixed at the time of exposure, then: *D = R × C × t *_*exp *_.

The ferret respiratory minute volume (V_m_), defined as the volume of air inhaled or exhaled over a minute, can be estimated using Guyton's formula [43], where BW = body weight in gr, and the volume calculated in ml:

The ferrets chosen for mock exposure studies ranged from about 500 to 900 gr. For a 500 gr ferret, log_10_BW^3/4 ^= 0.75 × log_10_500 = 2.02. The antilog of 2.02 = 105.7; therefore, V_m _= 2.10 × 105.7 = 222.0 ml/min (0.22 L/min). Similarly, for a 900 gr ferret, V_m _= 345.1 ml/min. Since most of the ferrets were close to 500 gr, an *approximate *V_m _value of 0.2 L/min was used for this work. The V_m _value of 0.2 L/min used in this work was consistent with estimates obtained by multiplying the ferret tidal volume (V_t_) expressed in ml × the breathing rate (BR) of conscious ferrets expressed as breaths/minute (bpm). By definition, V_t _= the volume of air inspired or expired with each normal breath, whereas BR = number of breaths/minute (bpm) for a conscious ferret. For ferrets, V_t _= 6.06 ± 0.30 ml, and BR = 33 - 36 bpm [44,45].

Using an average V_t _value of 6.06 ml and an average BR of 34.5 bpm, V_m _= 209.01 ml/min = 0.21 L/min.

For live agent work, the concentration of virus in the aerosol stream is estimated from the virus collected in impingers 1 and 2, where *Q *_*agi *__1+2 _is the collection flow rate in L/min through impingers (*agi *) 1 and 2, is:

The predicted respiratory volume during exposure (*V *_*e *_) is calculated as:

The aerosol concentration (*C *_*aero *_) needed to attain *D *is calculated as: *V *_*e *_*C *_*aero *_*= D *

## Competing interests

The authors declare that they have no competing interests.

## Authors' contributions

RST helped design and assemble the NBIES, established and protocols for inhalation exposure studies, helped with calculations, led efforts on the testing and validation of the NBIES, assisted with data interpretation, and co-wrote the manuscript; WAS assisted with the assembly, testing, and validation of the system, and oversaw animal work; DED assisted with program management, in-vitro virus work, and data interpretation; SBH established accurate virus quantification procedures, performed in-vitro virus work, and assisted with data interpretation; JAL conceived the overall NBIES design including choice of BANG nebulizer (with the assistance of all parties mentioned in the Acknowledgments section), interpreted data, established calculations, provided oversight, and co-prepared the manuscript. All authors read and approved the final manuscript.
